# Editorial: Novel Concepts in Using Broadly Neutralizing Antibodies for HIV-1 Treatment and Prevention

**DOI:** 10.3389/fimmu.2021.823576

**Published:** 2021-12-21

**Authors:** Kshitij Wagh, Marit J. van Gils, Harry Gristick, Philipp Schommers

**Affiliations:** ^1^ Theoretical Biology and Biophysics, Los Alamos National Laboratory, Los Alamos, NM, United States; ^2^ Department of Medical Microbiology and Infection Prevention, Amsterdam Institute for Infection and Immunity, Amsterdam University Medical Centers (UMC), University of Amsterdam, Amsterdam, Netherlands; ^3^ Division of Biology and Biological Engineering, California Institute of Technology, Pasadena, CA, United States; ^4^ Department of Internal Medicine, Faculty of Medicine and University Hospital Cologne, University of Cologne, Cologne, Germany; ^5^ German Center for Infection Research, Partner Site Bonn-Cologne, Cologne, Germany; ^6^ Laboratory of Experimental Immunology, Institute of Virology, Faculty of Medicine and University Hospital Cologne, University of Cologne, Cologne, Germany

**Keywords:** HIV, antibodies, acquired immunodeficiency syndrome (AIDS), Neutralization, bNAbs

## Introduction

Despite the success of antiretroviral therapy (ART) in suppressing HIV-1 replication and preventing disease progression, the high costs, the burden of daily medication, toxicity and the development of resistance underscore the need for new therapeutic approaches.

Over the past decade, broadly HIV-1 neutralizing antibodies (bNAbs) were discovered that are up to a 1000-fold more potent than HIV-1-reactive antibodies previously described. About 10 years after the first identification of these broadly neutralizing antibodies, bNAbs that effectively target multiple HIV-1 variants with a high potency have been found for most of the immunological important epitopes on the HIV-1 envelope-trimer like the CD4 binding site, the V1/V2 loop, the V3-glycan, the membrane-proximal external region (MPER), the interface region with the fusion peptide and the so called ‘silent face’. Some of these bNAbs have been demonstrated to safely suppress viremia and delay viral rebound after interruption of antiretroviral therapy (ART) in HIV-1-infected individuals. Moreover, bNAbs have been demonstrated to prevent infection in animal models and prevention studies where bNAbs are tested for their effectivity as passive immunization in humans are currently ongoing. Thus, bNAbs represent a promising novel approach for effective HIV-1 immunotherapy and prevention. However, infusions of single bNAbs drive the emergence of viral escape mutations and some patients harbor pre-existing resistance in their proviral or circulating HIV-1 quasispecies. Furthermore, the recently completed proof-of-concept Antibody Mediated Prevention (AMP) phase 2b trials showed that much higher bNAb titers or more potent and broader bNAbs, especially for single bNAbs, would be required for HIV-1 prevention in real-world settings. Thus, in order to restrict HIV-1 escape mechanisms and for improved antibody-mediated HIV-1 prevention, future regimens will require novel antibodies, antibody combinations or novel concepts like e.g. bi- or trispecific antibodies.

In this Research Topic, we aim to bring together new studies and comprehensive reviews that advance the field of bNAbs and their future clinical use for treatment and prevention of HIV-1.

## *In Vivo*/Clinical Studies

### Modeling HIV-1 Within-Host Dynamics After Passive Infusion of the Broadly Neutralizing Antibody VRC01

A better understanding of the effect of antibody treatment on viral loads will guide future antibody dosing strategies as well as gain insights on the mechanisms of viral clearance by the antibody. Cardozo-Ojeda and Perelson have used different mathematical models to explain the observed viral load dynamics in the VRC01 phase 1 clinical trial. A model containing reversible bNAb binding to virions and clearance of virus-antibody complexes by a two-step process explains best the observed viral loads. First, VRC01 induces an enhancement of virus clearance by a phagocytic mechanism, but due to saturation this process slows down and the long-term viral decline is due to neutralization. However, selection pressure may lead to the outgrowth of a less-susceptible virus population to VRC01 which is reflected in the viral load final rebound.

### Validation of a Triplex Pharmacokinetic Assay for Simultaneous Quantitation of HIV-1 Broadly Neutralizing Antibodies PGT121, PGDM1400, and VRC07-523-LS

An important clinical trial outcome is the accurate measurement of the passively infused bnAbs to determine effective doses for therapy and/or prevention. Wesley et al. describe an assay to simultaneously quantify the respective physiological concentrations of passively infused bnAb cocktails in human serum to ultimately define the threshold needed for protection from HIV-1 infection.

### Anti-Drug Antibodies in Pigtailed Macaques Receiving HIV Broadly Neutralising Antibody PGT121

Non-human primate models of passively transferred bNAbs for SHIV/SIV prevention and therapy have been critical for evaluating the *in vivo* bNAb activity. Nonetheless, the delivery of human-derived IgG in heterologous species such as rhesus macaques can limit their success due the animals developing antidrug antibodies (ADA) to human IgG. Such ADA responses restrict the number, frequency and doses of bNAbs given to non-human primates. Lee et al. extend these observations to the pigtailed macaque model. They show that such ADA responses were positively correlated with the number of doses and target the constant region of therapeutic bNAb, and not the variable region, resulting in cross-reactivity with either human control IgG1 antibody as well as another bNAb not delivered to the animals. Most notably, stronger ADA responses correlated with more precipitous decline of plasma bNAb concentrations and were significantly associated with worse control of simian HIV (SHIV). This study therefore outlines the caution that should be exercised in future studies of bNAb activity in pigtail macaques, and by extending the ADA observations to pigtail macaques, suggest that similar mechanisms could restrict study of bNAbs in other immunocompetent animal models.

### Characterizing the Relationship Between Neutralization Sensitivity and env Gene Diversity During ART Suppression

The diversity of replication competent HIV-1 latent proviruses and their susceptibility to therapeutic bNAbs are critical to successful bNAb-mediated HIV-1 therapy. Most HIV-1 infected induce robust autologous neutralizing antibodies (aNAbs) that drive viral Env escape, and this raises the following interesting questions: how do such autologous antibodies impact the composition of the latent reservoir, and how does escape from such autologous antibodies impact resistance to therapeutic bNAbs? Wilson et al. present compelling data present compelling data addressing these questions. They show that the latent reservoir can harbor aNAb resistant viruses and the latent viral Env diversity, presumably created by escape from aNAbs, can lead to resistance to certain therapeutic bNAbs, but not others. Clinical studies such as this can thus begin to address the key question of how many and which bNAbs will be needed to prevent viral breakthrough in analytical treatment interruption (ATI) studies and to ultimately succeed in HIV-1 therapy.

## *In Vitro* Studies

### Combinations of Single Chain Variable Fragments from HIV Broadly Neutralizing Antibodies demonstrate High Potency and Breadth

Single chain variable fragments (scFv) antibodies comprise of heavy and light chain variable fragments connected by glycine linkers in the same gene construct. Their smaller size as compared to full-length IgG can provide substantial advantages such as improved penetration of tissue, especially mucosa, and practical considerations such as expression by nucleic acids and viral vectors. However, the lack of Fc regions results in lower half-lives for scFv versus IgG and in the absence of antibody effector functions. The scFv molecules also lose some neutralization potency and breadth as compared to IgG due to loss of bivalent binding and/or subtly different paratope structures, albeit scFvs were shown to retain substantial activity of the parental antibodies. In this study, (van Dorsten et al.) explored whether combinations of scFvs targeting different HIV-1 Env epitopes can improve the breadth and potency against HIV-1 isolates. They show using experimental and theoretical approaches that combinations of scFv can significantly enhance breadth and potency over individual scFvs and that combinations of 2-3 scFvs could cover majority of viruses tested with high potency. By demonstrating that combinations of scFvs have favorably broad and potent *in vitro* neutralization profiles, this study lays the groundwork for further *in vivo* testing and development of scFv combinations as promising novel antibody-based prophylactics and therapeutics against HIV-1.

### HIV Broadly Neutralizing Antibodies Expressed as IgG3 Preserve Neutralization Potency and Show Improved Fc Effector Function

Richardson et al. describe the change in Fc-effector function profiles of HIV-1 bNAbs when expressed as IgG3 isotypes. Some HIV-1 reactive bnAbs expressed as IgG3 demonstrated enhanced binding to Fc receptors and improved FC-mediated effector functions while maintaining their neutralization activity. Thus, the antiviral effect of already very broad and potent bNAbs can even be enhanced by manipulations of the constant regions. These insights are important for the field given the current focus on using HIV-1 bNAbs for passive immunization strategies.

### Elimination of SHIV Infected Cells by Combinations of Bispecific HIVxCD3 DART^®^ Molecules

A potential future role of dual-affinity re-targeting Antibodies (DART) for treatment of HIV-1 is elegantly shown by Tuyishime et al. They investigated the effect of HIVxCD3 DART^®^ Molecules that have broadly-neutralizing and non-neutralizing activities on SHIV infected cells and found that these molecules effectively eliminated SHIV infected cells. Thus, these findings can be crucial for futures HIV-1 cure strategies since it is here shown that HIVxCD3 DART^®^ Molecules can leverage the host immune system for treatment of HIV-1 infection.

## Reviews

This Research Topic hosts articles that review currently available bNAbs and their potential future use. While Griffith and McCoy provide a comprehensive overview of the most promising currently available bNAbs, Hsu et al., Phelps and Balazs, and Umotoy and de Taeye review and outline the use of these bNAbs in future HIV-1 cure, treatment and prevention approaches.

### To bnAb or Not to bnAb: Defining Broadly Neutralising Antibodies Against HIV-1

Griffith and McCoy describe a possible definition of antibody features that are required by a specific antibody to be classified as “broadly neutralizing”. To this end they review and compare the neutralizing profiles, mutations, genetic features and the targeted epitopes of an array of currently known neutralizing antibodies. Through this effort the authors provide a comprehensive and unbiased overview of the currently available bNAbs of which several are or will be tested in clinical studies.

### Can Broadly Neutralizing HIV-1 Antibodies Help Achieve an ART-Free Remission?

In this review Hsu et al. concisely and yet thoroughly review the potential role of HIV bNAbs in strategies to achieve long term virologic control in the absence of ART. The authors comprehensively review this complicated area of research with clinical studies from many different groups. They discuss a plethora of preclinical and clinical studies that aim to determine if bNAbs can act alone, in combination, and/or together with other drugs to control HIV infection and eliminate or decrease the viral reservoir.

### Contribution to HIV Prevention and Treatment by Antibody- Mediated Effector Function and Advances in Broadly Neutralizing Antibody Delivery by Vectored Immunoprophylaxis

While neutralization is a crucial component of therapeutic efficacy, numerous studies have demonstrated that bNAbs can also mediate effector functions. Phelps and Balazs review key concepts of effector functions mediated by bNAbs and the potential for vectored immunoprophylaxis as a means of producing bNAbs in patients to overcome the need for constant re-administration in order to maintain steady-state bNAb concentrations.

### Antibody Conjugates for Targeted Therapy Against HIV-1 as an Emerging Tool for HIV-1 Cure

By reading the review of Umotoy and de Taeye readers will get a thorough and historical perspective on the use antibody conjugates (ACs) in HIV research. The authors describe the concept of an antibody-based carrier of anti-HIV-molecules and why specifically bNAbs could be the ideal carrier. The anti-HIV-molecules include toxins fused to the antibody, antibodies conjugated by radionuclides (radioimmunotherapy) and small drugs or oligonucleotides conjugated to antibodies. In contrast to anticancer treatment, for which several constructs are already FDA-approved, no AC is currently approved for treatment of viral infections. However, with new potent bNAbs available ACs have a huge potential for future HIV-1 cure strategies which is discussed in detail by the authors.

### Broadly Neutralizing Antibodies for HIV-1 Prevention

The current status of clinical studies evaluating bNAbs for HIV-1 prevention is reviewed by Walsh and Seaman. To this end, the authors summarize key clinical trials like the recently published AMP-trial, based on their advantage and how they contributed to the field. Besides recent clinical studies, the authors also discuss strategies that will lead to an improved breadth, potency and durability of antiviral protection in future clinical trials.

## Conclusion and Outlook

Altogether, bNAbs show promise for successful HIV-1 prevention, therapeutic control and potential towards HIV-1 cure, especially when they are further tailored to improve breadth, potency, bioavailability and increased killing of infected cells through protein engineering. For future evaluation of bNAb prophylaxis and therapy in clinical trials it is crucial to have well validated quantitative assays with well-predicting antibody distribution and viral load models.

## Author Contributions

All authors listed have made a substantial, direct, and intellectual contribution to the work and approved it for publication.

## Funding

KW was supported by the Comprehensive Antibody Vaccine Immune Monitoring Consortium (CAVIMC) (Grant: OPP1032144 from the Bill & Melinda Gates Foundation.

## Conflict of Interest

The authors declare that the research was conducted in the absence of any commercial or financial relationships that could be construed as a potential conflict of interest.

## Publisher’s Note

All claims expressed in this article are solely those of the authors and do not necessarily represent those of their affiliated organizations, or those of the publisher, the editors and the reviewers. Any product that may be evaluated in this article, or claim that may be made by its manufacturer, is not guaranteed or endorsed by the publisher.

